# Gut Steroids and Microbiota: Effect of Gonadectomy and Sex

**DOI:** 10.3390/biom12060767

**Published:** 2022-05-31

**Authors:** Silvia Diviccaro, Jamie A. FitzGerald, Lucia Cioffi, Eva Falvo, Fiona Crispie, Paul D. Cotter, Siobhain M. O’Mahony, Silvia Giatti, Donatella Caruso, Roberto Cosimo Melcangi

**Affiliations:** 1 Department of Pharmacological and Biomolecular Sciences, University of Milano, Via Balzaretti 9, 20133 Milano, Italy; silvia.diviccaro@unimi.it (S.D.); lucia.cioffi@unimi.it (L.C.); eva.falvo@unimi.it (E.F.); silvia.giatti@unimi.it (S.G.); donatella.caruso@unimi.it (D.C.); 2 Teagasc Food Research Centre, Moorepark, P61 C996 Cork, Ireland; jamie.fitzgerald@teagasc.ie (J.A.F.); fiona.crispie@teagasc.ie (F.C.); paul.cotter@teagasc.ie (P.D.C.); 3 APC Microbiome Ireland, University College Cork, College Road, T12 YT20 Cork, Ireland; somahony@ucc.ie

**Keywords:** pregnenolone, sex steroids, gut microbiota, branched- and short-chain fatty acids, sex dimorphism, gastrointestinal tract, mucosa, stool

## Abstract

Sex steroids, derived mainly from gonads, can shape microbiota composition; however, the impact of gonadectomy and sex on steroid production in the gut (i.e., gut steroids), and its interaction with microbiota composition, needs to be clarified. In this study, steroid environment and gut steroidogenesis were analysed by liquid chromatography tandem mass spectrometry and expression analyses. Gut microbiota composition as branched- and short-chain fatty acids were determined by 16S rRNA gene sequence analysis and gas chromatography flame ionisation detection, respectively. Here, we first demonstrated that levels of pregnenolone (PREG), progesterone (PROG), and isoallopregnanolone (ISOALLO) were higher in the female rat colon, whereas the level of testosterone (T) was higher in males. Sexual dimorphism on gut steroidogenesis is also reported after gonadectomy. Sex, and more significantly, gonadectomy, affects microbiota composition. We noted that a number of taxa and inferred metabolic pathways were associated with gut steroids, such as positive associations between *Blautia* with T, dihydroprogesterone (DHP), and allopregnanolone (ALLO), whereas negative associations were noted between *Roseburia* and T, ALLO, PREG, ISOALLO, DHP, and PROG. In conclusion, this study highlights the novel sex-specific association between microbiota and gut steroids with possible relevance for the gut-brain axis.

## 1. Introduction

The gut microbiota is a community of bacteria, archaea, viruses, and fungi located in the gastrointestinal tract [1,2,3,4] that contributes to host homeostasis, both in regulating immunity and metabolism, as well as protection against pathogens [5,6]. Among several factors which can influence the composition of the gut microbiota, sex is a major contributor [7]. Both human and animal studies, mainly performed in mice, have reported sex-related differences in the gut microbiota [8,9,10,11,12,13,14], and a role has been proposed for sex steroid hormones in contributing to this sexual dimorphism [9,12,13,14,15,16,17,18,19]. Additionally, inhibition of key enzymes in steroidogenesis, such as 5-alpha-reductase (5α-R), alters gut microbiota populations in rodents and humans [20,21]. On the other hand, gut microbes also regulate the availability of sex steroids in the gut environment, such as oestrogens [22,23,24,25,26,27] and androgens [26,28,29]. Microbiota and their derived metabolites actively participate in host homeostasis via the gut-brain axis, a bi-directional communication highway [26,30,31,32,33,34], which includes immune, endocrine, neural, and humoral routes [31,35,36]. 

Sexual dimorphism in the levels of steroids derived from gonads, as well as those synthesized in the nervous system (i.e., neurosteroids), has also been previously reported [37,38]. In the context of functional interaction between gut microbiota and sex steroids, it is important to highlight that these molecules may also be directly synthesized in the gut. Indeed, as recently observed, the adult male rat colon expresses steroid molecules involved in the early steps of steroidogenesis, and in the consecutive synthesis and metabolism of sex steroids, such as progesterone (PROG), testosterone (T), and 17-beta-estradiol (17β-E) [39]. In particular, several enzymes involved in steroidogenesis are expressed in the adult male rat colon [39]: steroidogenic acute regulatory protein (StAR), responsible for the transfer of cholesterol into mitochondria where the first step of the steroidogenesis occurs; cytochrome P450 cholesterol side-chain cleavage enzyme (P450scc), responsible for the conversion of cholesterol into pregnenolone (PREG); 3β-hydroxysteroid dehydrogenase (3β-HSD), responsible for the conversion of PREG into PROG; 5α-R and 3α-hydroxysteroid oxidoreductase (3α-HSOR), involved in the generation of active metabolites of PROG such as dihydroprogesterone (DHP) and allopregnanolone (ALLO), as well as metabolites of T, such as dihydrotestosterone (DHT) and 5α-androstane-3α, 17β-diol (3α-diol); and aromatase, an enzyme which converts T into 17β-E. Interestingly, the receptor for luteinizing hormones (i.e., the gonadotropin responsible for steroidogenesis) is not only expressed in the gonads [40], but also in the gastrointestinal tract of humans and rats [41,42,43,44]. Local steroidogenesis was also supported by the finding that significant levels of these steroids were assessed in the colon of male rats, and that the gut levels were higher than those present in the plasma [39].

On this basis, here we evaluate (1) if gut steroidogenesis shows sexually dimorphic features; (2) the sex contribution of peripheral steroid hormones in gut steroidogenesis; (3) the sexually dimorphic features of gut microbiota and both branched- and short-chain fatty acids (BCFA and SCFA), as well as their correlations with steroid levels present in the rat gut. In particular, the levels of free PREG and its sulphate form, PROG and its derivatives (i.e., DHP, ALLO and isoallopregnanolone, ISOALLO), dehydroepiandrosterone (DHEA), T and its derivatives (i.e., DHT, 3α-diol and 17β-E) were evaluated in control and gonadectomized (i.e., one month of gonadectomy) male and female rats by liquid chromatography tandem mass spectrometry. The expression of enzymes and molecules related to steroidogenesis was also assessed. Gut microbiota composition and BCFA/SCFA were determined by 16S rRNA gene sequence analysis and gas chromatography flame ionisation detection, respectively. 

## 2. Materials and Methods

### 2.1. Animals

Adult male and female Sprague Dawley rats, both intact (control) and gonadectomized (GDX), were purchased from Charles River Laboratories, Lecco, Italy. Animals were housed in the animal care facility of the Dipartimento di Scienze Farmacologiche e Biomolecolari (DiSFeB) at the Università degli Studi di Milano, Italy. All animals were pair-housed, in standard cages, with a regular diet (‘chow’, grain based; ssniff Spezialdiäten GmbH, Soest, Germany), tap water available ad libitum, and under a controlled humidity and temperature. The procedures were performed in accordance with national (D.L. No. 26, 4 March 2014, G.U. No. 61, 14 March 2014) and international laws and policies (EEC Council Directive 2010/63, 22 September 2010: Guide for the Care and Use of Laboratory Animals, United States National Research Council, 2011), and were previously approved by the local ethics committee, and by the Italian Ministry of Health (authorization 1083/2015-PR). 

### 2.2. Study Design

Intact females were monitored via a vaginal swab to establish the oestrus cycle. The oestrous cycle lasts four days in female rats and is characterized as: proestrus, oestrus, metestrus, and diestrus, which may be determined according to the cell types observed in the vaginal smear. The vaginal secretion of rats was collected with a swab every morning and was observed using the microscope. Control female rats were sacrificed during the diestrus cycle. The animals GDX were pair-housed in an animal facility for 1 month after the surgery. Control and GDX rats were sacrificed at the same age (i.e., 3 months old). 

### 2.3. Sample Collection

Faecal samples were collected the afternoon before the day of sacrifice for BCFA/SCFAs and microbiome analysis; they were immediately frozen in liquid nitrogen, transported to the laboratory, and stored at −80 °C. At sacrifice, the animals were individually placed in an induction chamber, and anaesthesia was given with 2% isoflurane (ISO VET, La Zootecnica, Milan, Italy) until loss of righting reflex. Superficial mucosa was separated from lamina propria, with the aid of a scraper, and collected in 0.5 mL of saline solution (NaCl 0.9%). Mucosa and segments of the colon were harvested and immediately frozen in liquid nitrogen and stored at −80 °C until the analyses. 

### 2.4. Liquid Chromatography Tandem Mass Spectrometry Analysis (LC–MS/MS) for Steroids 

For quantitative analysis of steroids, longitudinal sections of the colon (200 mg/sample) were collected, and internal standards, such as 17β-Estradiol-2,3,4-^13^C_3_ (2 ng/sample), progesterone-2,3,4,20,25^13^C_5_-PROG (0.4 ng/sample), and pregnenolone-20,21-^13^C_2_-16,16 D_2_ (10 ng/sample), were added. The tissue was processed as previously described [39]. Briefly, the colon tissues were homogenized using the Tissue Lyser (Qiagen, Milan, Italy), in ice cold MeOH/Acetic acid 1% and purified by organic phase extraction as previously described [45]. The quantitative analysis was performed using a linear ion trap-mass spectrometer (LTQ, Thermo Fisher Scientific, Waltham, MA, USA) equipped with a Surveyor liquid chromatography (LC) Pump Plus and a Surveyor Autosampler Plus (Thermo Fisher Scientific, Waltham, MA, USA), operating in APCI positive mode. Chromatographic separation was achieved on an Hypersil Gold column C18 (100 × 2.1 mm, 3 μm, Thermo Fisher Scientific, Waltham, MA, USA). LC–MS/MS data were evaluated using Excalibur^®^ release 2.0 SR2 (Thermo Fisher Scientific, Waltham, MA, USA). Quantitative analysis of PREG, PREG sulphate, PROG, DHP, ALLO, ISOALLO, DHEA, T, DHT, 3α-diol, and 17β-E were achieved based on calibration curves which were freshly prepared.

### 2.5. Assessment of Steroidogenic Machinery 

#### 2.5.1. Quantitative Real-Time PCR 

RNA was extracted from the snap-frozen colon using Direct-zolTM RNA MiniPrep kit (Zymo Research, Irvine, CA, USA) following the manufacturers protocol, after homogenization in the Tissue Lyser (Qiagen, Italy) with EUROGOLD TriFast (Euroclone, Milano, Italy). The quantification of RNA was performed by NanoDropTM 2000 (ThermoFisher scientific, Milano, Italy). After quantification, gene expression was assessed by TaqMan quantitative real-time PCR using a CFX96 real-time system (Bio-Rad Laboratories, Milan, Italy). Briefly, the total RNA from each sample (10 or 150 μg) were run in 96-well formats in duplicate as multiplexed reactions with a normalizing internal control, 36B4 (Eurofins MWG-Operon, Milano, Italy), using the Luna Universal One-Step RT-qPCR Kit (New England BioLabs Inc., Ipswich, MA, USA). Specific TaqMan MGB probes and primer sequences were purchased at Eurofins MWG-Operon (Milano, Italy) and are available on request: StAR, 5α-R1, 5α-R2, 3α-HSOR, HSL, ACAT1, STS, SULT2β1. Specific TaqMan MGB probes (Applied Biosystems, Thermo Fisher Scientific) were: CYP11A1 (Rn00568733_m1) and 3β-HSD (Rn00820880_g1). 

#### 2.5.2. Western Blotting

For Western blotting analysis, the colon was homogenized using the Tissue Lyser (Qiagen, Italy) in the lysis buffer (PBS without Ca^2+^ and Mg^2+^, EDTA 0.5 M pH 8, Igepal), supplemented with a protease cocktail inhibitor (Roche Diagnostic spa, Monza, Italy). Then, to remove particulate matter, the colon homogenate was centrifugated at 2000 rpm for 5 min at 4 °C. The colon tissue was centrifugated a second time if required and then the supernatant was analysed. The protein content of colon lysate was quantified using a Bradford Assay (Bio-Rad, Milan, Italy) and samples containing equal amounts of protein were heated to 100 °C for 5 min. Samples were run on Criterion TGX 4–15% gradient gels (Bio-Rad, Milan, Italy) and transferred to nitrocellulose membranes. Stain-free technology (Bio-Rad, Milan, Italy) was used to determine equal loading. Nitrocellulose membranes were blocked in 5% bovine serum albumin (BSA) for 1 h at room temperature and exposed to primary antibodies of aromatase (ab191093, abcam 1:500). After incubation, membranes were then washed for 1 h and then incubated with an anti-rabbit horseradish peroxidase conjugated secondary antibody (1:1000). The protein bands were detected on membranes using the ECL method (Bio-Rad, Milan, Italy). ECL signals were acquired with a ChemiDocTM XRS+ system (Bio-Rad, Milan, Italy) and analysed with Image LabTM software version 5.2.1 (Bio-Rad, Milan, Italy). The detected target proteins were normalized with the total lane values obtained with the stain-free technology (Bio-Rad, Milan, Italy). The mean control value within a single experiment was set to 100.

### 2.6. Short-Chain Fatty Acid Analysis

SCFAs analysis was carried out on samples collected from the faeces of each rat as previously described [46]. Formic acid, acetic acid, propionic acid, iso-butyric acid, butyric acid, iso-valeric acid, valeric acid, hydrochloric acid and 2-ethyl-butyric acid (internal standard, IS) were purchased from Sigma Aldrich. An IS stock solution of 100 mM in formic acid and external standard solution of acetate (20 mM), propionate (20 mM), Iso-butyrate (2 mM), butyrate (20 Mm), Iso-valerate (2 mM) and valerate (20 mM) in water were generated and stored at −20 °C. A standard curve (acetate, propionate, butyrate and valerate at 0.1 mM, 0.5 mM, 1 mM, 2 mM, 4 mM, 8 mM, and 10 mM; isobutyrate and isovalerate at 0.01 mM, 0.05 mM, 0.1 mM, 0.2 mM, 0.4 mM, 0.8 mM, and 1 m) was generated and run before samples were analysed. The IS was added to both samples and standards at 1 mM. Frozen faecal samples were homogenised by vortexing them with milli-Q water (1:10 *w*/*v*) for 10 min with the pH reduced to 2.5 with the addition of concentrated HCl. The solutions were centrifuged at 20,000× *g* for 15 min at 4 °C. The supernatant was syringe filtered (0.22 μm, Corning) and a 270 μL aliquot was mixed with 30 μL of 10 mM of IS in duplicate. The samples were vortexed and then centrifuged at 20,000× *g* before being transferred to 250 μL glass inserts with polymer feet (Agilent) and placed in amber glass 2 mL GC vials (Agilent), and sealed with silicone/PTFE screw caps (Agilent).

#### Gas Chromatography Flame Ionisation Detection Analysis

Standards and samples were analysed using gas chromatography flame ionisation detection (GC-FID) using a Varian 3800 GC system, fitted with a DB-FFAP column (30 mL × 0.32 mm ID × 0.25 μm df; Agilent) and a flame ionisation detector. Samples and standards were loaded (0.2 μL splitless injection) with a CP-8400 autosampler (Agilent). Helium was employed as the carrier gas at a flow rate of 1.3 mL/min. The initial GC oven temperature was set at 50 °C, and was maintained for 0.5 min, raised to 140 °C at 10 °C/min and held for 0.5 min, before being increased to 240 °C at 20 °C/min, and held for 5.0 min (total run time 20 min). The temperatures of the detector and the injection port were set at 300 °C and 240 °C, respectively. Peaks were integrated using Varian Star Chromatography Workstation version 6.0 software. Blank water vials were analysed between each sample duplicate to check for any potential carryover.

### 2.7. 16S rRNA Gene Sequence for Microbiota Analysis

Faecal and mucosal samples for microbiota analysis were processed as described previously [47]. Briefly, faecal samples for microbiota analysis were homogenized and processed using mechanical and chemical lysis. DNA was extracted using the QIAamp PowerFecal Pro DNA Kit (QIAGEN). DNA concentration was normalized and 16S metagenomic libraries were prepared using primers to amplify the V3–V4 region of the bacterial 16S rRNA gene (S-D-Bact-0341-b-S-17, S-D-Bact-0785-a-A-21; expected product length of 464 bp), with Illumina adaptors incorporated as described in the Illumina 16S Metagenomic Library Preparation guide. Following index PCR and purification, the products were quantified using the Qubit high sensitivity DNA kit (Life Technologies, Milan, Italy) and pooled at equimolar concentrations. The pooled libraries were assessed using an Agilent high sensitivity DNA kit and quantified by quantitative PCR (qPCR) using the Kapa Quantification kit for Illumina (Kapa Biosystems, Wilmington, MA, USA) according to the manufacturer’s guidelines. Libraries were then diluted and denatured following Illumina platform guidelines and sequenced (2 × 300 bp) on the Illumina MiSeq platform.

### 2.8. Bioinformatics

Sixty-four samples were sequenced (mean of 151,290, SE of 12,289 reads per sample) alongside a negative sequencing control. Primer sequences and Illumina adapter sequences were queried via Cutadapt to remove sequencing artefacts (−m of 200, −e of 0.2) [48]. Sequence quality was supervised via FastQC and MultiQC [49,50]. FIGARO [51] determined per-sample optimal trimming and filtering parameters (sequence truncation lengths and maximum expected errors) which were then decreased by 1 to increase stringency. These values were then supplied to DADA2 (parameters: 3 × 10^8^ bases for error profiling, no variant pooling, a minimum read-pair overlap of 20 bp and mismatch of 0) [52] with consensus removal of bimeras, and taxonomic identity was assigned via DADA2’s assignSpeciesTaxonomy function, using the SILVA taxonomic reference database [53,54], in order to determine the presence and abundance of unique amplicon sequencing variants (ASVs) representative of the different microbial populations.

Off-target ASVs (product length <400 bp, >426 bp), ASVs lacking taxonomic assignment at the phylum level, and single-copy ASVs were removed, giving 1301 ASVs in total. Samples with a total read abundance of less than 500 were dropped from further consideration, leaving 63 samples. A negative sequencing control showed negligible contamination (55 reads, 4 Clostridia taxa) and was also removed. Finally, to produce a dataset addressing the likely metabolic potential of the microbiome community characterized here, ASV abundances were used to predict samples’ metabolic pathway abundances MetaCyc [55] using PICRUSt2 [56] with default parameters.

### 2.9. Statistical Analysis

Data obtained by LC-MS/MS were analysed using a two-way analysis of variance (ANOVA), with sex and gonadectomy as two independent variables, followed by the Tukey post-hoc test. Data obtained by real time PCR were analysed by Student’s *t* test. *p* ≤ 0.05 was considered significant. All analyses were performed using GraphPad PRISM (La Jolla, CA, USA) (version 7.2). Alpha diversity (observed species, Shannon’s H, Inverse Simpson’s index, and Faith’s phylogenetic index) was estimated across a rarefied range of sampling depths (100 to 10,000 reads, 100 iterations, R library rtk [57]), and tested using linear mixed-effect models (R library lme4) [58] to account for repeated sampling (stool, mucosa) of the same animal, with a false discovery correction rate of 0.05. Differences in beta diversity (Jaccard dissimilarity from relative abundances) between experimental group, sex, and substrate were tested via PERMANOVA (function adonis) [59]. Constrained correspondence analysis models (function cca) were iterated using the step.cca function [59] to identify the most significant environmental contributions of community composition. 

### 2.10. Differential Abundance Testing

Feature abundances (ASVs, predicted metabolic pathway abundances) were standardized for library size using the R library GMPR [60] and transformed using centre-log ratios (CLR) [61], before filtering to exclude features not abundant above 0.5% in 10% of samples. Feature-wise differences in CLR abundance between experimental, gender, and substrate groups were tested using linear mixed-effect models (lme4 package, R) [58] to account for repeated sampling of the same subject. Significant differences in estimated mean abundances (R library emmeans) [62] were filtered using a false discovery control rate of 0.05, and plotted (ggplot, r-base) [63,64].

### 2.11. Correlations among Diversity/Microbiome with SCFA and Steroids

The relationships between steroids (PREG, PREG sulphate, PROG, DHP, ALLO, ISOALLO, DHEA, T, DHT, 3⍺-diol and 17β-E), BCFA (isobutyrate, isovalerate), SCFA (acetate, propionate, butyrate and valerate), alpha diversity (Shannon’s H) and the microbiome were evaluated for interplay between the effects of sex, gonadectomy, and the microbiome. 

To balance the number of steroid/FA samples per animal (1 each) with the number of microbiome samples per animal (2 each; stool, mucosa), mucosal samples were omitted when considering the relationship between microbiome and steroidogenesis/FA metabolism. ASVs and EC pathways, filtered and transformed as above for differential testing, were correlated with steroid and SCFA concentrations (Spearman’s ρ; bootstrapped *p* values with 1000 iterations) as a means of visualizing the broader relationship between the microbiome and steroid/FA abundance. Significant feature-parameter associations (*p* < 0.05) were retained and plotted via heatmap (ComplexHeatmap) [65], using sample-wise z-scoring of abundances.

## 3. Results

### 3.1. Assessment of Steroid Levels

**Figure 1:**
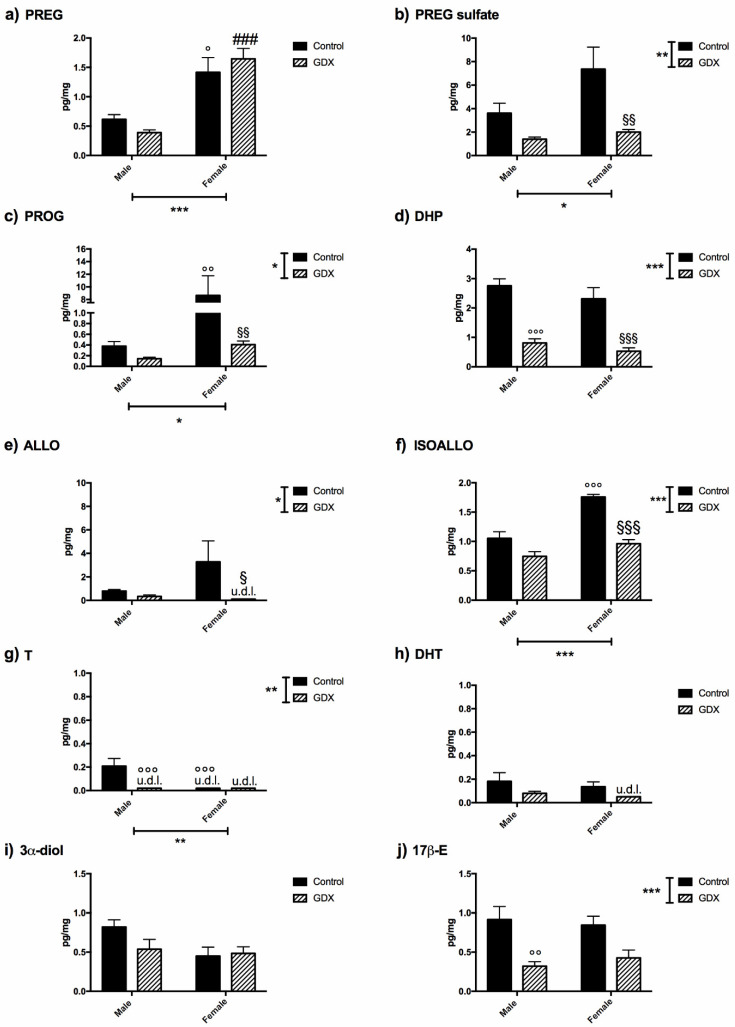
Steroid levels assessed by LC-MS/MS in the colons of male or female gonadectomized (GDX) rats compared with a sham-operated control. The panels represent the levels of (**a**) pregnenolone (PREG); (**b**) pregnenolone sulfate (PREG sulfate); (**c**) progesterone (PROG); (**d**) dihydroprogesterone (DHP); (**e**) allopregnanolone (ALLO); (**f**) isoallopregnanolone (ISOALLO); (**g**) testosterone (T); (**h**) dihydrotestosterone (DHT); (**i**) 5α-androstane-3α, 17β-diol (3α-diol); (**j**) 17β-estradiol (17β-E). Data are expressed as pg/mg and are the mean ± SEM. *n* = 6 animals for each experimental group. u.d.l. = under detection limit. Limit of detection for ALLO is 0.1 pg/mg, for T is 0.02 pg/mg and for DHT is 0.05 pg/mg. The two-way ANOVA was used for statistical analysis. * *p* < 0.05, ** *p* < 0.01, *** *p* < 0.001. The multiple comparison Tukey post-hoc test was used. ° *p* < 0.05, °° *p* < 0.01, °°° *p* < 0.001 vs. male control group; ### *p* < 0.001 vs. male GDX group; § *p* < 0.05, §§ *p* < 0.01, §§§ *p* < 0.001 vs. female control group.

The levels of several steroids were assessed using LC–MS/MS in the colons of a control female (i.e., on the day of dioestrus), control male, GDX female, and GDX male rat. Two-way ANOVA analysis was performed using sex and gonadectomy as two independent variables, followed by the Tukey multiple comparison post-hoc test. As reported in Figure 1a, the level of PREG showed a significant effect of sex (F1,20 = 40.92, *p* < 0.0001).

Indeed, as revealed by the Tukey post-hoc test, the levels of this steroid were significantly higher in the control female vs. control male rats (*p* = 0.0105) as well as in GDX female rats vs. GDX male rats (*p* = 0.0001). The levels of PREG sulphate (Figure 1b) showed a significant effect of sex (F1,20 = 4.381, *p* = 0.0493) and gonadectomy (F1,20 = 13.27, *p* = 0.0016). In particular, the levels of this steroid were significantly lower in GDX female rats vs. control female rats (*p* = 0.0081). 

The levels of the first metabolite of PREG, PROG (Figure 1c), were also significantly different depending on sex (F1,20 = 7.508, *p* = 0.0126) and gonadectomy (F1,20 = 7.412, *p* = 0.0131), and interaction was noted between these factors (F1,20 = 6.608, *p* = 0.0182). The Tukey post-hoc test revealed that the PROG levels were significantly higher in control female animals compared with male animals (*p* = 0.0063). Interestingly, PROG levels were significantly decreased after gonadectomy in females (*p* = 0.0065) but not in male rats due to significantly lower levels of PROG in males at baseline. 

The levels of the 5α-reduced metabolite of PROG and DHP (Figure 1d) were only affected by gonadectomy (F1,20 = 59.12, *p* < 0.0001), and were significantly lower in both male (*p* < 0.0001) and female (*p* = 0.0002) GDX animals compared with their controls. Similarly to DHP, the levels of its 3α-5α-reduced metabolite, ALLO (Figure 1e) also showed a significant overall effect of gonadectomy (F1,22 = 4.824, *p* = 0.0389); however, when assessed by sex, a decrease in the levels of ALLO was observed only in GDX female rats (*p* = 0.0473). 

The colonic levels of 3β-5α-reduced metabolite of DHP, ISOALLO (Figure 1f), was significantly affected by sex (F1,20 = 33.09, *p* < 0.0001), gonadectomy (F1,20 = 47.21, *p* < 0.0001), and an interaction between the factors was noted (F1,20 = 9.241, *p* = 0.0065). As observed in the case of PROG levels, the Tukey post-hoc test revealed that ISOALLO levels were significantly higher in the control female vs. control male animals (*p* < 0.0001), and that only in females was ALLO significantly decreased after gonadectomy (*p* < 0.0001). Although the levels of DHEA, precursor of androgens, were below the detection limit (0.050 pg/mg) in all experimental groups, significant levels of T were detected in control male rats (Figure 1g). A two-way ANOVA indicated that T levels were significantly impacted by sex (F1,24 = 11.54, *p* = 0.0024) and gonadectomy (F1,24 = 11.54, *p* = 0.0024), and here again, an interaction was seen (F1,24 = 11.54, *p* = 0.0024) (Figure 1g). T levels in control male rats were significantly higher vs. GDX male animals (*p* = 0.0004) and vs. control female animals (*p* = 0.0008). No differences were detected in the levels of the T metabolites DHT (Figure 1h) and 3α-diol (Figure 1i), but for 17β-E (Figure 1j), the two-way ANOVA revealed a significant effect of gonadectomy (F1,24 = 19.24, *p* = 0.0003). In particular, the Tukey post-hoc test revealed a significant decrease of 17β-E levels in GDX male vs. control male animals (*p* = 0.0081).

### 3.2. Gut Steroidogenic Machinery

**Figure 2:**
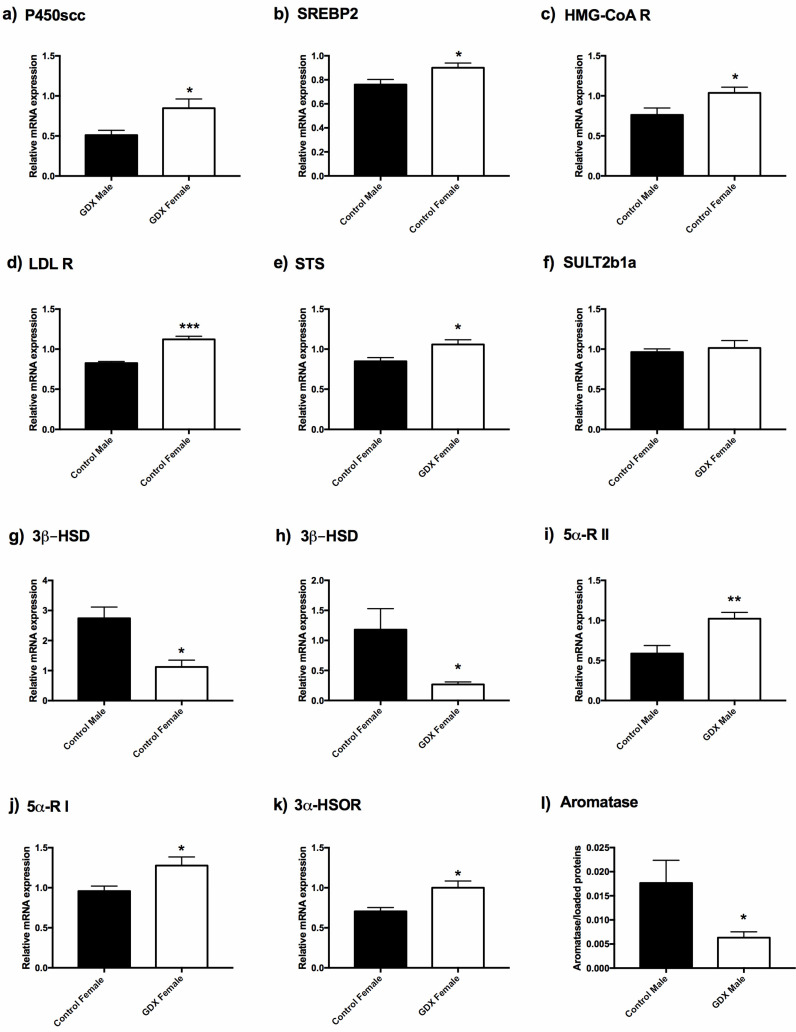
Expression of enzymes detected in rat colons. Data were obtained by real time PCR in panels (**a**) cholesterol side-chain cleavage enzyme (P450scc); (**b**) sterol regulatory element-binding protein 2 (SREBP2); (**c**) 3-hydroxy-3-methylglutaryl-coenzyme A reductase (HMG-CoA R); (**d**) low-density lipoprotein receptor (LDL R); (**e**) steroid sulfatase (STS); (**f**) hydroxysteroid sulfotransferase (SULT2B1); (**g**,**h**) 3β-hydroxysteroid dehydrogenase (3β-HSD); (**i**) 5α-reductase type II (5α-R II); (**j**) 5α-reductase type I (5α-R I); (**k**) 3α-hydroxysteroid-oxidoreductase (3α-HSOR), and by Western blot analysis in panel (**l**) aromatase. The columns represent the mean ± SEM after normalization with 36B4 rRNA in colon of adult rats. *n* = 6 animals for each experimental group. The unpaired Student’s *t*-test was used for statistical analysis. * *p* < 0.05, ** *p* < 0.01, *** *p* < 0.001.

On the basis of data obtained by LC–MS/MS, we examined whether the effect of sex and gonadectomy on steroid levels could be associated with the changes in the expression levels of genes involved in sex steroid synthesis within the colon. As depicted in Figure 1a, the levels of the precursor of sex steroids, PREG, are higher in the control and GDX females than in males. This sex dimorphic feature might be due to (1) increased production of the enzyme responsible for its synthesis (i.e., P450scc) and/or (2) higher cholesterol bioavailability. Data obtained indicated that the gene expression of P450scc was similar in control females vs. control males (Female: *n* = 6, 0.458 ± 0.102 vs. male: *n* = 6, 0.720 ± 0.104) but was higher in GDX females vs. GDX males (Figure 2a). 

As is evidenced by Figure 2b, gene expression levels of sterol regulatory element-binding protein 2 (SREBP2), which is involved in the regulation of the biosynthesis of cholesterol, were higher in control females vs. control males, but not in GDX animals (GDX female: *n* = 6, 0.912 ± 0.027 vs. GDX male: *n* = 6, 0.942 ± 0.060). SREBP2 binds and specifically activates 3-hydroxy-3-methylglutaryl-coenzyme A reductase (HMG-CoA R) and the low-density lipoprotein receptor (LDL R). HMG-CoA R converts 3-hydroxy-3-methyl-glutaryl-coenzyme A to mevalonate (the rate limiting step in cholesterol biosynthesis) whereas LDL R internalizes cholesterol-rich lipoproteins as LDL into the cellular compartment. In accordance with the demonstration of the highest SREBP2 expression, the gene expression of HMG-CoA R (Figure 2c) and LDL R (Figure 2d) were also significantly higher in control females than in control males. As portrayed in Figure 1b, gonadectomy induced, in female animals, a significant decrease in PREG sulphate levels. Accordingly, the gene expression of steroid sulfatase (STS) [66], the enzyme converting PREG sulphate into free PREG, was upregulated (Figure 2e), whereas that of hydroxysteroid sulfotransferase (SULT2B1a), the enzyme which catalyses the sulfation of PREG [66], was unchanged in control females vs. GDX females (Figure 2f).

As depicted in Figure 1c, the levels of PROG were higher in control females vs. control males and were significantly decreased by gonadectomy in female animals. Accordingly, the gene expression of 3β-HSD (i.e., the enzyme converting PREG to PROG) was significantly decreased by gonadectomy in female animals (Figure 2h); however, contrary to the levels of PROG, the gene expression of this enzyme was higher in male than in female control animals (Figure 2g). 

Gonadectomy also affects DHP levels, however in this case, the decrease in steroid levels occurred in both sexes (Figure 1d). Assessment of two isoforms of the enzyme 5α-R showed that in male animals, gonadectomy induced an increase in the gene expression of type 2 5α-R (Figure 2i), whereas that of type 1 was unaffected (control male: *n* = 6, 0.812 ± 0.070 vs. GDX male: *n* = 6, 0.962 ± 0.097). On the contrary, in female animals, the gonadectomy induced an increase in the gene expression of type 1 (Figure 2j) but not of the type 2 enzyme (control female: *n* = 6, 0.737 ± 0.194 vs. GDX female: *n* = 6, 0.822 ± 0.192). Gonadectomy also decreased the levels of the further metabolite, ALLO, in female animals only (Figure 1e). Gene expression for the enzyme responsible for its formation, 3α-HSOR (Figure 2k), was upregulated by gonadectomy. Finally, as shown in Figure 2l, the protein levels of aromatase (i.e., the enzyme converting T into 17β-E) was, as indicated by the levels of 17β-E (Figure 1j), decreased in GDX males vs. controls. 

### 3.3. Microbial Diversity

**Figure 3:**
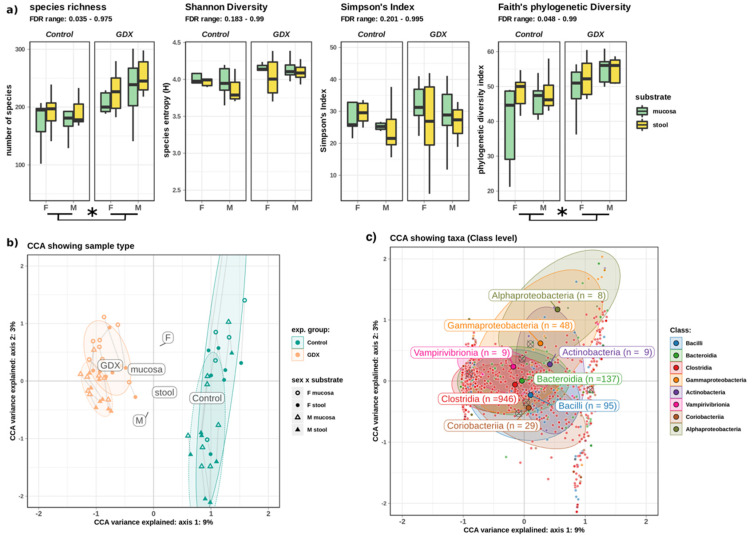
Microbial diversity. (**a**) Alpha diversity (richness and phylogenetic diversity) was increased following gonadectomy, but it did not differ significantly between sexes or substrates. (**b**) Beta diversity (CCA of sample composition), showing the significant influence of both experimental group and sex, as illustrated by the strong separation of labels (centroids) for F and M controls, which is resolved by GDX. The substrate had a non-significant effect, as illustrated by the co-location of labels near the origin. (**c**) The same CCA showed taxa as points closest to samples where they were most abundant (e.g., *Gammaproteobacteria* tended to be more abundant in control-F). Crossed circles indicate location of group centroids. Paired samples are joined by a line. Asterisk (*) denotes significant difference at FDR < 0.05, whereas a horizontal bracket indicates that the difference is between the GDX and control overall. Abbreviations: F: female; M: male; GDX: gonadectomy; CCA: constrained correspondence analysis.

Significant increases in alpha diversity were noted following gonadectomy (Figure 3a), specifically in species richness and Faith’s phylogenetic measures of alpha diversity (*p* < 0.01). 

Although no significant differences in alpha or beta diversity were observed between mucosal and stool samples, experimental group and sex exerted a strong, highly significant effect on the composition of the microbiome (PERMANOVA of Jaccard’s dissimilarity, *p* < 0.0001). Constrained correspondence analysis (CCA) of microbial composition indicated that experimental group (control, GDX) had the strongest effect on composition, whereas sex contributed a smaller, but significant component (Figure 3b). These effects partially bifurcated the microbial community, with many taxa most abundant in either the control or GDX rats (Figure 3c). 

### 3.4. Abundance of Taxa

**Figure 4:**
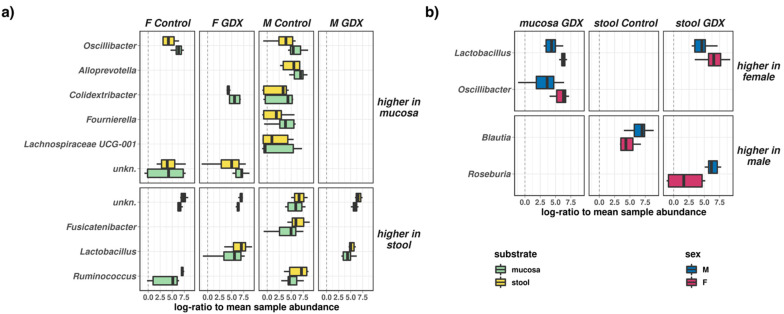
Differential effects of sex and substrate on the abundances of individual microbial genera. (**a**) Taxa which were differentially abundant (FDR < 0.05) between mucosal and stool samples for different groups across the experimental design. (**b**) Taxa which were differentially abundant (FDR < 0.05) between female and male samples for different groups across the experimental design.

Next, we independently characterised the effects of sex on the abundance of individual microbial taxa in both mucosa and stool samples (i.e., substrates) in our study. We noted several significant (FDR ~0.001~0.05, Figure 4a) changes in microbiome composition due to substrate. 

Some taxa were more abundant in mucosal samples (*Oscillibacter*: higher in mucosa of control females and males; *Colidextribacter*: higher in mucosa of GDX females and control males; *Alloprevotella*, *Fournierella*, and *Lachnospiraceae* UCG-001: higher in mucosa of control males), whereas others were more abundant in stool samples (*Ruminococcus*: higher in stool of control females and males; *Lactobacillus*: higher in stool of GDX females and males; *Fusicatenibacter*: higher in stool of control males). We also noted limited differences (FDR 0.02–0.04, Figure 4b) between the female and male microbiomes: *Oscillibacter* (mucosa-GDX) and *Lactobacillus* (mucosa-GDX, stool-GDX) were significantly more abundant in female samples, whereas *Blautia* (stool-control) and *Roseburia* (stool-GDX) were more abundant in samples from males.

**Figure 5:**
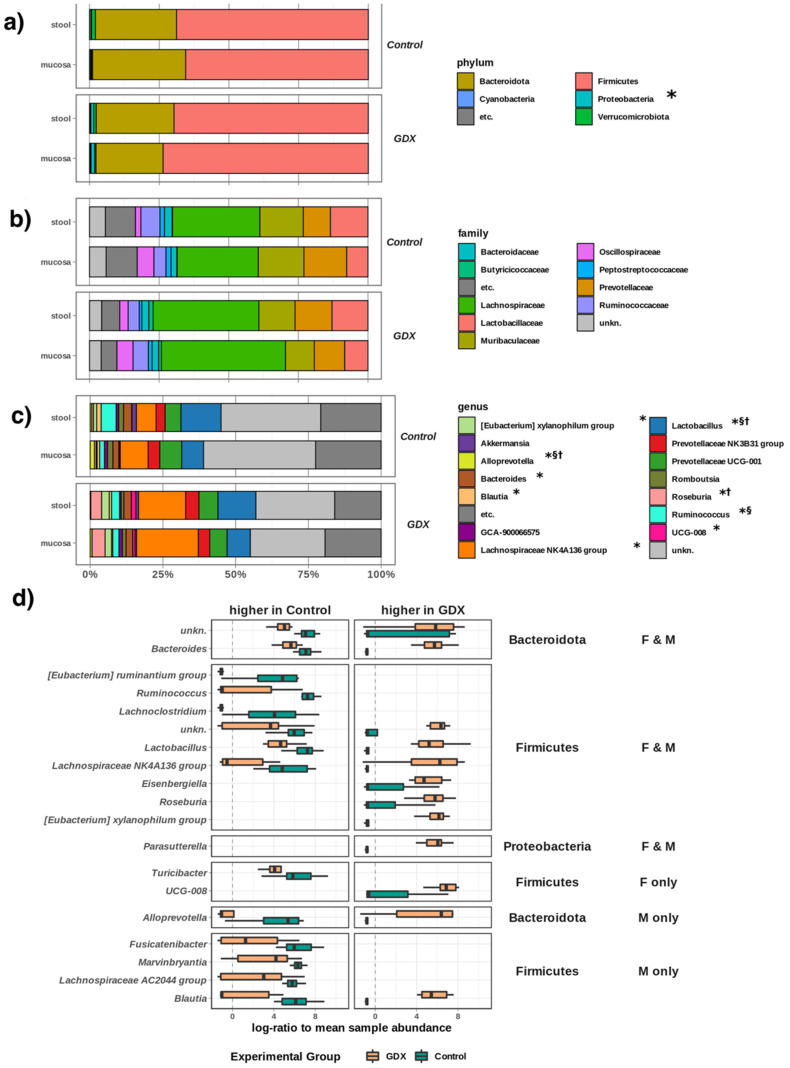
Microbial composition and taxa significantly affected gonadectomy: (**a**): Phylum level; (**b**): Family level; (**c**): Genus level; (**d**): Summary of genera which differ significantly across the study (FDR < 0.05), whether in both females (F) and males (M), F only, or M only. Some genera (e.g., *Bacteroides*, *Alloprevotella*) appear higher in both the control and GDX rats as there are multiple distinct members (e.g., species) within that genus, which show different responses to sex and GDX. (**a**–**c**): all taxa > 3.5% abundance, in 10% of samples (remainder grouped to “etc.”). “*” indicates taxa differentially abundant between experimental groups (GDX, control); “†” indicates taxa differentially abundant between substrates (mucosa, stool); “§” indicates differentially abundant taxa between sexes (male, female).

Having characterised underlying microbiome differences in the experimental design, we next considered the effect of gonadectomy on the stool and mucosal microbiome of both males and females. There was no experimental effect on the overall abundances of *Firmicutes* and *Bacteroides*, indicating that gonadectomy led to specific alterations in the compositions of these major groups, rather than a gross alteration of the dominant phyla; however, we noted that overall abundance of the phylum *Proteobacteria* was significantly increased following gonadectomy (both male and female, *p* < 0.001, Figure 5a). 

There were no significant differences in abundance between control and GDX rats at the family level (Figure 5b); only at the genus-level did significant changes in composition become apparent (Figure 5c,d), with significant decreases in abundance in both GDX females and males for the *Lachnospiraceae* ‘NK4A136′ group, *Bacteroides*, *Ruminococcus*, *Lachnoclostridium*, and *Eubacterium ‘ruminantium’* genera, as well as several uncharacterised taxa. Although there were decreases in multiple taxa between *Firmicutes* (*Fusicatenibacter*, *Marvinbryantia*, *Lachnospiraceae* AC2044 group, *Blautia*) and *Bacteroidetes* (*Alloprevotella*) in GDX males, only one genus-level decrease was specific to GDX females (*Firmicutes*: *Turicibacter*), indicating a stronger sex-specific effect of GDX on the microbiome in males. 

In addition to significant decreases in relative abundance to some taxa, multiple genera increased in abundance post-gonadectomy in both sexes (*Bacteroides*, *Lactobacillus*, *Lacnospiraceae NK4A136*, *Eisenbergiella*, *Roseburia*, *Xylanophilum*, and *Parasutterella* genera). Interestingly, the significant decreases in *Blautia* and *Alloprevotella*, which, as mentioned above, are specific to GDX males, appear to be compensated for, with significant increases in alternate taxa from the *Blautia* and *Alloprevotella* genera in GDX males only (Figure 5d). Only the uncharacterised group, ‘UCG-008′, increased in GDX females, again indicating the low level of sex-specific effects of GDX on the female microbiome relative to males.

### 3.5. Short-Chain Fatty Acids and Branched-Chain Fatty Acids

**Figure 6:**
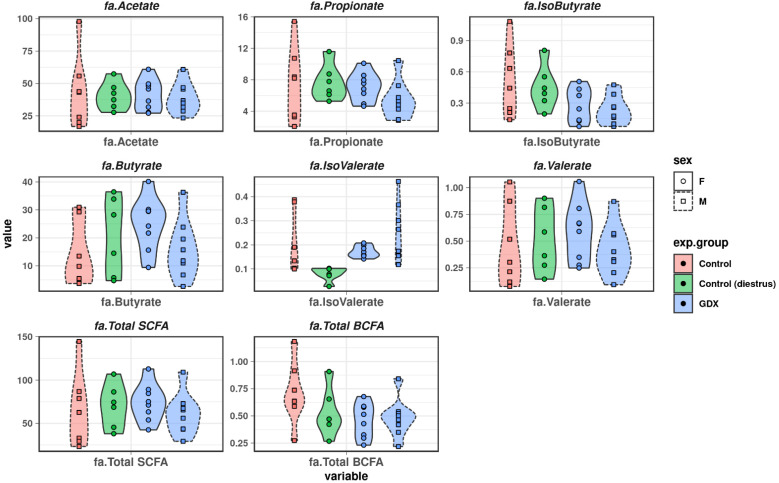
Faecal short-chain fatty acid and branched chain fatty acid levels in all groups.

We characterised levels of SCFA and BCFA across control and GDX male and female groups. SCFA, such as acetate, butyrate, and propionate comprised the majority of fatty acids detected (Figure 6), with low levels of BCFA (isobutyrate, isovalerate). 

Although there were some apparent differences in levels of SCFA and BCFA (elevated butyrate in control and GDX females; elevated isovalerate in control and GDX males; lower levels of isobutyrate in GDX females and GDX males), we found no statistically significant differences between males and females, or in levels induced by gonadectomy (Figure 6). 

### 3.6. Interactions between Taxa and Steroidogenesis

**Figure 7:**
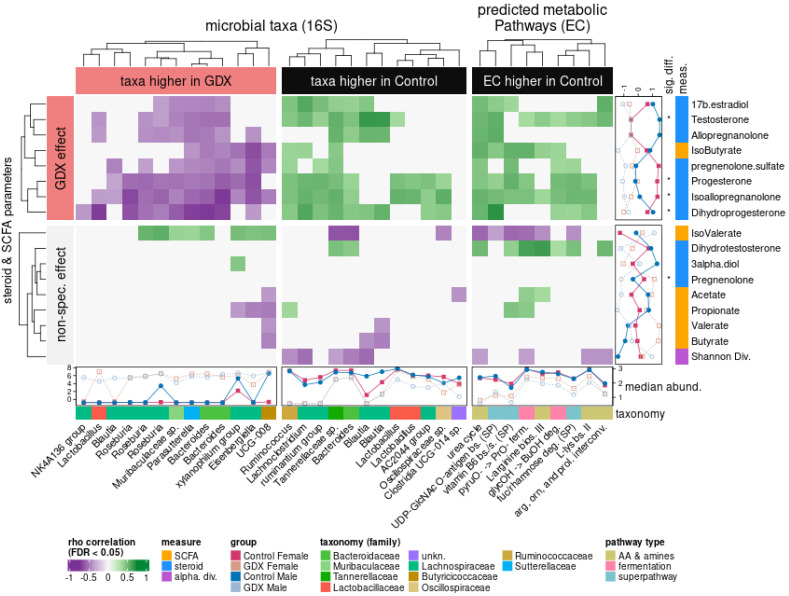
Microbiome features associated with control are positively correlated (green) with an abundance of multiple steroids. This also extends to predicted metabolism, which only shows positive correlations with steroid levels; many of these steroids are also significantly more abundant in the control rats (*, Figure 2). This unified pattern suggests that the control gut microbiome metabolizes a range of host steroids and is negatively affected when these substrates are depleted by gonadectomy. Disruption via GDX favours an alternate cohort of bacteria, but it does not foster new predicted metabolic activities. Heatmap: colours reflect positive (green) or negative (purple) Spearman’s ρ correlations between microbial features (ASVs, predicted ECPs and biological parameters: steroids, fatty acids, Shannon’s alpha diversity). Top row (GDX effect): summarizes associations relevant to experimental design; bottom row (non-spec. effect): summarizes incidental associations which do not cluster with the main experimental effect. Right-hand margin: unit-scaled abundances, significant differences if applicable (*), and categories for biological parameters in this study (steroid, fatty acids, alpha diversity). Bottom margin: scaled abundances, and microbial taxonomy (ASVs) or pathway types (ECPs) of features. All microbial features (ASV, EC) shown here were found to differ significantly between control and GDX (FDR < 0.05).

Having independently identified both sex steroids and taxa affected by gonadectomy, we hypothesised that the intervention would also allow correlation of metabolic interactions between steroids, BCFA/SCFA, and the gut microbiome. We combined all three datasets (microbial, steroids, and fatty acids), as well as a dataset of predicted metabolic pathway (EC) abundances derived from the observed composition of microbial samples. Bootstrapped correlations between significantly different microbial features (taxa, predicted pathways) and environmental parameters (steroids, BCFA/SCFA, alpha diversity) illustrated two broad effects of the experimental design (Figure 7): firstly, the effect of GDX revealed both positive and negative correlations between the microbiome and host steroids. 

As hormone levels were generally higher in control groups, the primary effect of gonadectomy could be further subdivided into microbes positively correlated with higher steroid levels in the control rats (Figure 7, green), and microbes negatively correlated with steroid levels, leading to a higher microbial abundance in the GDX rats (Figure 7, purple). The second broad effect was an association of microbes to gut parameters (BCFA/SCFA values and Shannon’s alpha diversity index, H) not specifically arising from the experimental contrast, but rather from observations across the dataset (Figure 7, “non-spec. effect”).

Some differences in microbial changes between the GDX and control rats appeared ‘matched’, with distinct, alternate (e.g., *Lactobacillus* and *Bacteroides*) features that were more abundant in either the GDX or control rats, thus suggesting a change in the niche conditions as a result of the intervention, leading to a substitution of closely-related species. In contrast, there are many genera that appear more abundant in either one case or the other: e.g., several *Blautia* taxa are more abundant in control and positively associated with T, DHP, and ALLO, whereas *Roseburia* correlated negatively with ALLO, ISOALLO, DHP, PROG, and T.

Interestingly, predicted metabolic pathways only showed positive correlations with steroid levels, suggesting that a range of microbial activities (amino acid and amine metabolism, fermentation, and catabolic/anabolic superpathways) interact with, or are reliant upon, host hormone levels under normal conditions (positive correlations in control), but that these interactions are obviated in the GDX rats, and are not coherently replaced by the microbiome. The strongest correlation for predicted microbial function was between the UDP-N-acetyl-D-glucosamine superpathway and DHP (rho = 0.698), which also correlated with DHT (rho = 0.348), whereas other metabolisms (fermentation, amines, and amino acids) ranged from rho = 0.34–0.653. Steroids which differed significantly between experimental groups (Figure 1, and see “*” notation in Figure 7) represented the steroids most frequently associated with the microbiome, with the exception of PREG, which, although significantly different in terms of abundance across the study, was not associated with any taxon or predicted pathway. 

Although isovalerate was not significantly more abundant in the GDX rats, several GDX-associated taxa were correlated positively with it, whereas taxa correlated with control showed negative correlations. Shannon diversity was also negatively associated with a number of control-associated features, which follows from the observation of increased alpha diversity and more variable beta diversity in the GDX rats (Figure 3), thus reflecting a post-gonadectomy gut microbiome that is significantly more variable. 

Taken together, these changes in the microbiome suggest a large background shift in composition as a result of gonadectomy, likely due to the restriction of steroid availability as a substrate, as well as a number of sex-specific changes in the microbiome after gonadectomy.

## 4. Discussion

**Figure 8:**
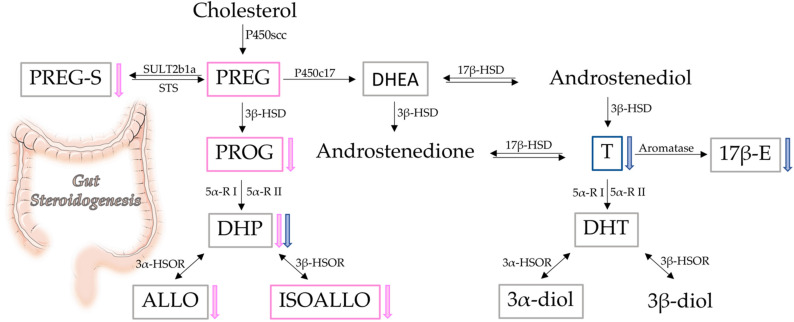
Schematic representation of gut steroidogenesis. Framed steroids reported have been assessed by LC-MS/MS. Pink frame: higher levels in intact female; blue frame: higher levels in intact male; grey frame: no sex difference. Arrows represent the effect of gonadectomy in female (pink) and in male (blue).

Steroids, microbiota, BCFA/SCFA, and their interrelationships were investigated in the colons of male and female rats in this study. Here, we extend our previous results, indicating that not only male [39], but also female rat colons, possess steroidogenic capability. Interestingly, the levels of some steroids show a sexually dimorphic pattern in the colon. Indeed, as summarized in Figure 8, the levels of PREG, PROG, and ISOALLO were higher in females, whereas the levels of T were higher in males. As reported above, the sexually dimorphic pattern of PREG was confirmed by the analysis of gene expression of molecules related to this steroid. In particular, we reported that the higher levels of PREG in females are not associated with higher gene expression of the enzyme synthesizing this steroid (i.e., P450scc), the concentrations of which were similar between female and male colon tissue. Rather, the genes involved in synthesis (i.e., SREBP2, HMG-CoA R and LDL R) of the primary substrate for P450scc, cholesterol, were found to be more highly expressed in control females than control males, indicating that increased availability of cholesterol is responsible for the higher levels of PREG.

The finding that both male and female rat colons possess steroidogenic capability is also supported by the observations obtained in gonadectomized animals. In the GDX group, decreases in T, DHP, and 17β-E levels in GDX males and decreases in PREG sulphate, PROG, DHP, ALLO, and ISOALLO in GDX females, were reported (Figure 8). Although this endocrine manipulation generally decreased circulating levels of these molecules, meaningful levels of sex steroids persisted in the colon, with the exception of T in males. The observed decrease in 17β-E levels in the GDX male colon was in agreement with the low bioavailability of aromatase (i.e., the enzyme converting T into 17β-E) observed in this tissue. The reported decrease in PREG sulphate is of particular interest in the context of the unchanged levels of PREG observed in females after gonadectomy. Notably, free PREG is in equilibrium with its sulphate form by the action of the enzyme SULT2B1a, which is responsible for the sulfation of PREG, and by the enzyme STS, which retro-converts PREG sulphate into free PREG [67]. As reported here, gonadectomy in females increased the expression of the enzyme STS, providing a mechanism for the decrease of PREG sulphate, and consequently, the maintenance of free PREG levels. Furthermore, gene expression levels for enzymes related to metabolism of DHP and ALLO also appeared to compensate for decreased levels of these steroids as a result of gonadectomy. Indeed, we here reported that the gene expression of the 5-alpha-reductase (i.e., enzyme converting PROG into DHP) [68] was upregulated in a sexually dimorphic way, depending on the isoforms considered. Gonadectomy induced an increase in the gene expression of type 1 only in females, and of type 2 only in males. Additionally, the gene expression of 3α-HSOR (i.e., the enzyme converting DHP into ALLO) was upregulated by gonadectomy in female rat colons.

In the context of gut physiology, and consequently of its local steroidogenesis, it is also important to consider the impact on the central nervous system, potentially through the gut-brain axis [31,35,36]. The nervous system can also locally synthesize steroids (i.e., neurosteroids) in a sexually dimorphic way [38], and it adapts its steroid levels in response to changes in gonadal steroid hormones [45]; however, the sexually dimorphic pattern of neurosteroids does not mirror that of the pattern noted in the gut. For instance, in contrast to what was previously reported regarding the colon, PREG and PROG levels are higher in male brain areas, such as the cerebral cortex and cerebellum [45]. Additionally, the levels of some steroids are sexually dimorphic in the brain, but not in the colon (e.g., higher levels of DHP and ALLO observed in female brain areas) [45]. Furthermore, and at variance to observations here obtained in the colon, gonadectomy induced a significant increase in PREG levels in areas of the female brain, including the cerebral cortex and cerebellum, whereas gonadectomy of males led to a decrease in ALLO in the same brain locations [69]. In this context, it is important to highlight that the effects of gonadectomy on steroidogenic enzymes may be different between the two compartments. For instance, in contrast to what we previously observed in the female colon after gonadectomy, expression of the gene 3α-HSOR was seen to decrease in the cerebellum of female GDX rats [70]. Altogether, these results suggest that steroidogenesis along the gut-brain axis is affected differently by sex and peripheral steroid hormones, depending on the tissue considered. 

Data obtained reveal that rat gonadectomy significantly altered the gut microbiome, increasing alpha diversity and defining beta diversity (i.e., community composition) with a greater effect than either sex or sample substrate. Taxonomic analysis further indicated significant changes in microbial abundances, with differences in both mucosal and stool samples. Microbiota in the mucosa is closer to the intestinal epithelium, and it may interact more with the host than the faecal bacteria. A model has been proposed to explain an oxygen gradient existing within the intestinal environment, from aerobic (i.e., mucosal interface) to anaerobic (i.e., intestinal lumen) in a steady-state environment [71]; however, when the intestinal barrier breaks down, aerotolerant members of the mucosal microbiome are capable of translocating across the epithelial barrier surface. Here, we observed a higher Proteobacteria abundance in the mucosal substrate compared to stool samples in control rats (Figure 5A), albeit this difference is obviated through gonadectomy. Interestingly, in both sexes, *Parasutterella* (phylum: *Proteobacteria*) significantly increase after gonadectomy, and it is negatively correlated with several steroids, in particular, DHP (Figure 6). Increases in the same Proteobacteria genus are also frequently associated with irritable bowel disease and inflammatory bowel disease [72], suggesting that depletion of local sex steroids might represent ecological stress in a manner similar to inflammation of the intestinal environment. The abundances of some taxa appeared sex-linked in the microbiota community. Indeed, in GDX females, we observed higher *Oscillibacter* in mucosa and *Lactobacillus* in mucosa and stools; in control males we noted higher *Blautia* in stools; and in GDX males, we noted higher *Roseburia* in stools. For instance, mucosal samples showed higher *Oscillibacter*, *Colidextribacter*, *Alloprevotella*, *Fournierella*, *Lachnospiraceae UCG-001* in male controls, and higher *Oscillibacter* and *Colidextribacter* in female control and GDX females, respectively. Stool samples showed higher abundances of taxa such as *Ruminococcus* and *Fusicatenibacter* in male controls, *Lactobacillus* in GDX males and females, and *Ruminococcus* in female control. At the genus levels, decreases in *Lachnospiraceae*, *Bacteroides*, *Ruminococcus*, and *Lachnoclostridium* in male and female GDX rats, *Fusicatanibacterium*, *Marvinbryantia*, *Blautia*, *Alloprevotella* and Lactobacillus in male GDX rats, and *Turicibacter* in female GDX rats was reported. In contrast, increases in *Lactobacillus*, *Lacnospiraceae NK4A136*, *Eisenbergiella*, *Roseburia*, *Bacteroides*, the *Eubacterium xylanophilum* group, and *Parasutterella* were reported for male and female GDX rats, *Blautia* and *Alloprevotella* for male GDX rats, and *uncharacterized UCG-008* in female GDX rats. Altogether, this set of analyses confirmed and extended the previous literature data indicating sex differences in gut microbiota [8,9,10,11,12,13,14] and the role of sex steroid hormones [9,12,14,15,16]. As described above, the gut exhibits sexual dimorphism in both its steroidogenic capability and microbial composition. In addition, we have characterized the interactions between taxa and gut steroids, showing that taxa such as *Blautia* were more abundant in control animals and were positively associated with T, DHP, and ALLO, whereas *Roseburia* and others were negatively correlated with T, ALLO, PREG, ISOALLO, DHP, and PROG. 

Although this study also characterized the levels of BCFA/SCFA, no significant effect of gonadectomy on the abundance of these metabolites was found. Accordingly, levels of BCFA/SCFA showed little interaction with the GDX-associated microbiome, as evident by the placement of most FAs in the cluster demarked as “non-specific effects” (Figure 7). The exception to this is iso-butyrate, which was more abundant in the control, and correlated with microbial features impacted by gonadectomy. Although butyrate is a key metabolite in gut health [73,74], much less is known about the role of the branched isomer isobutyrate. As we demonstrated here, this BCFA was positively associated with *Lachnoclostridium* and *uncharacterised Tannerellaceae*, and correlated negatively with *Bacteroides*, *Parasutterella*, *Eisenbergiella*, and other uncharacterized taxa. Although they are also positively correlated with predicted pathways (urea, UDP, glycerol deg, v B6, pyruvate fermentation, L-arginine bios), the levels of isobutyrate detected in this study were low, requiring additional characterization. 

Our analysis of steroid levels also reported exclusively positive correlations with the predicted metabolic pathways, indicative of metabolic activity reliant on normal sex steroid levels. In particular, the strongest correlation was between DHP levels in control animals and UDP-N-acetyl-D-glucosamine, which was also associated with many other steroids including T and DHT. The lipopoly-saccharide UDP-N-acetyl-D-glucosamine forms an essential precursor of cell wall peptidoglycan, and represents a common enterobacterial antigen [75], whereas UDP (uridine di-phosphate) is used ubiquitously as a structural molecule and substrate for sugar metabolism, and is also used as a solubility promoter for transport of conjugated sex steroids (e.g., T, DHT) [76,77]. Although the gut microbiome has been shown to activate T and DHT through the de-conjugation of associated UDP [28], the gut microbiome was not characterized, and no mechanism was demonstrated. In this work, emphasis falls upon the strongest positive correlation between UDP-N-acetyl-D-glucosamine pathways and DHP. To our knowledge, specific observations on DHP are not present in the literature; however, it is worth noting that some PROG metabolites have been observed undergoing glucuronidation in several forms [78]. Therefore, it is possible that DHP acts as a substrate for UDP metabolism as documented in the case of T and DHT, facilitating microbial uptake of UDP for the UDP-N-acetyl-D-glucosamine pathway. Although this metabolic pathway appears inhibited in GDX rats, it contributed to the differences in community composition; however, an applied and direct characterization of the microbial metabolism is required to test this hypothesis.

## 5. Conclusions

Altogether, observations here reported show that local steroidogenesis in the rat colon and its microbiota are different in the two sexes and affected by the peripheral sex steroid hormones. The interactions between the local steroidogenesis and gut microbiota may have pathophysiological relevance not only for the gut itself, but also in the context of the gut/brain axis. Indeed, it is well known that many gut [79,80], as well as nervous disorders [81,82,83,84], exhibit sexual dimorphism.
